# Enteral Glutamine Administration in Critically Ill Nonseptic Patients Does Not Trigger Arginine Synthesis

**DOI:** 10.1155/2016/1373060

**Published:** 2016-04-20

**Authors:** Mechteld A. R. Vermeulen, Saskia J. H. Brinkmann, Nikki Buijs, Albertus Beishuizen, Pierre M. Bet, Alexander P. J. Houdijk, Johannes B. van Goudoever, Paul A. M. van Leeuwen

**Affiliations:** ^1^Department of Internal Medicine, VU University Medical Center, 1081 HV Amsterdam, Netherlands; ^2^Department of Surgery, VU University Medical Center, 1081 HV Amsterdam, Netherlands; ^3^Department of Intensive Care Unit, VU University Medical Center, 1081 HV Amsterdam, Netherlands; ^4^Intensive Care Unit, Medisch Spectrum Twente, 7511 JX Enschede, Netherlands; ^5^Department of Clinical Pharmacology and Pharmacy, VU University Medical Center, 1081 HV Amsterdam, Netherlands; ^6^Department of Surgery, Medical Center Alkmaar and Trial Center Holland Health, Alkmaar, Netherlands; ^7^Department of Pediatrics, VU University Medical Center, 1081 HV Amsterdam, Netherlands; ^8^Department of Pediatrics, Emma Children's Hospital, AMC, 1105 AZ Amsterdam, Netherlands

## Abstract

Glutamine supplementation in specific groups of critically ill patients results in favourable clinical outcome. Enhancement of citrulline and arginine synthesis by glutamine could serve as a potential mechanism. However, while receiving optimal enteral nutrition, uptake and enteral metabolism of glutamine in critically ill patients remain unknown. Therefore we investigated the effect of a therapeutically relevant dose of L-glutamine on synthesis of L-citrulline and subsequent L-arginine in this group. Ten versus ten critically ill patients receiving full enteral nutrition, or isocaloric isonitrogenous enteral nutrition including 0.5 g/kg L-alanyl-L-glutamine, were studied using stable isotopes. A cross-over design using intravenous and enteral tracers enabled splanchnic extraction (SE) calculations. Endogenous rate of appearance and SE of glutamine citrulline and arginine was not different (SE controls versus alanyl-glutamine: glutamine 48 and 48%, citrulline 33 versus 45%, and arginine 45 versus 42%). Turnover from glutamine to citrulline and arginine was not higher in glutamine-administered patients. In critically ill nonseptic patients receiving adequate nutrition and a relevant dose of glutamine there was no extra citrulline or arginine synthesis and glutamine SE was not increased. This suggests that for arginine synthesis enhancement there is no need for an additional dose of glutamine when this population is adequately fed. This trial is registered with NTR2285.

## 1. Introduction

Previously, numerous clinical studies demonstrated that supplementation with glutamine as free molecule or dipeptide results in a favourable clinical outcome as reflected by a reduction in infectious morbidity (trauma [1, 2] and medical [2–5] patients), mortality [3, 6], and a reduction in length of hospital stay in severely ill patients [4, 7–9]. However, the use of high dose glutamine in shock patients has been part of debate [10]. The underpinning mechanism of the clinical effects of exogenous glutamine administration has not been completely elucidated yet. The effects of glutamine could be partially explained by the substrate that glutamine is for the synthesis of citrulline and arginine. Citrulline may act as a radical scavenger and is also a potent arginine precursor [11]. Arginine is of great importance for wound healing and the immune system and it is the precursor of nitric oxide (NO) [12–16]. During trauma and sepsis, plasma concentrations of arginine are decreased [17]. However, the action of arginine as a substrate for nitric oxide synthesis with potential subsequent hemodynamic instability and oxidative stress may be responsible for the reported adverse events of glutamine administration in severe critically ill patients [18–20]. Since endogenous glutamine can generate arginine by the citrulline pathway in the kidney, supplying glutamine may be a more physiologic and safe way to regulate arginine availability in the metabolically stressed ICU patient [21, 22]. However, in critically ill patients the metabolic fate of glutamine is still unclear. Possibly, generally altered metabolism could exist due to impaired enterocyte function because of injury, splanchnic ischemia, sepsis, and starvation.

Considering the observation that the gut preferentially takes up enterally provided glutamine, with subsequent higher intestinal release of citrulline, the precursor for arginine, we decided to provide L-alanyl-L-glutamine by the enteral route in this study, expecting to deliver glutamine most adequately to the interorgan pathway of glutamine into citrulline and arginine [23, 24]. Hence the objective of this clinical study was to investigate the effect of the enteral administration of a clinically relevant dose of L-glutamine, provided as L-alanyl-L-glutamine, on the synthesis of L-citrulline from L-glutamine and the subsequent synthesis of L-arginine from citrulline in critically ill nonseptic patients. Quantitatively, tracer methodology was used to determine exact turnover of these amino acids. A cross-over study design using intravenous and enteral tracers was chosen enabling splanchnic extraction calculations. We hypothesized that after enteral glutamine supply splanchnic glutamine uptake would increase as well as subsequent increases in citrulline and arginine synthesis.

## 2. Patients and Methods

### 2.1. Patients

Twenty critically ill patients considered stable were studied. All were expected to stay at the ICU for at least 5 days. Additional inclusion criteria were age, ≥18 years, BMI ≥ 18.5 and ≤35, ability to tolerate enteral nutrition, provided by postpyloric tube, meeting full protein/energy requirements based on indirect calorimetric measurements, and a protein intake of 1.2–1.7 g/kg/day.

Exclusion criteria were septic shock (defined according to the International Guidelines for Diagnosis of Sepsis [25]); need for high dose vasoactive medication such as norepinephrine higher than 0.2 *μ*g/kg/min; PaO_2_/FiO_2_ ratio < 200; PEEP > 15 cm H_2_O; liver failure (bilirubin levels > 100 *μ*mol/L); hyperammonaemia (ammonia > 50 *μ*mol/L); kidney failure (renal replacement therapy or increase in serum creatinine levels to >100 *μ*mol/L), in the absence of primary underlying renal disease, associated with oliguria (defined as urine output < 150 mL in the previous 8 hours); urea cycle defects; chronic corticosteroids use (>7.5 mg/day >3 weeks); gastrointestinal malabsorption possibly interfering with intestinal absorptive function (celiac disease, Crohn's disease, presence of fistulas, major intestinal malabsorption disorder, or short bowel syndrome); pregnancy or lactation; admission after elective surgery; parenteral nutrition; use of medium chain triglycerides or glutamine/citrulline supplements.

Informed consent was obtained from all included patients or his/her legal representative. The Medical Ethical Committee of the VU University Medical Hospital approved the study protocol (MEtC VUmc 2009.083). The study complied with the Declaration of Helsinki (NTR2285).

### 2.2. Study Design

**Figure 1:**
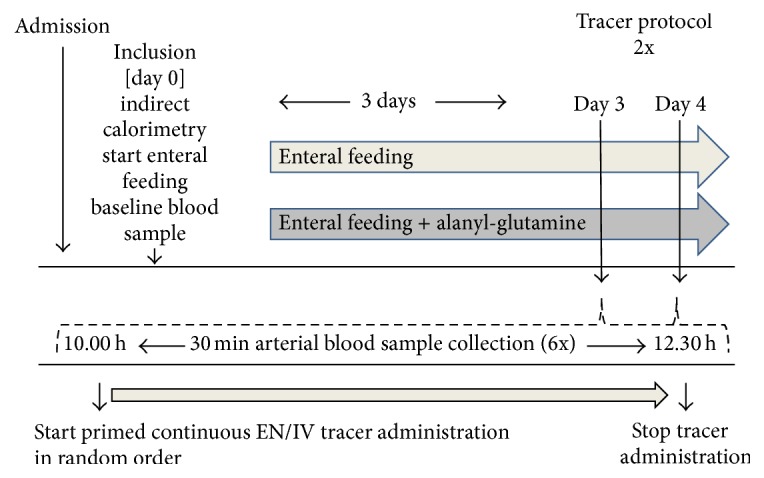
Study protocol.

All twenty patients received enteral nutrition via postpyloric or nasogastric tube. Ten patients received an additional enteral dose of 0.5 g/kg/day L-alanyl-L-glutamine (ALA-GLN) (=0.325 g/kg glutamine/day) (Fresenius Kabi Nederland B.V. Den Bosch, Netherlands). Patients in the control group received isonitrogenous enteral nutrition without the additional glutamine (CON). Total nitrogen was compensated by using different enteral nutritional formulas containing different amounts of protein. Patients were investigated while being fed continuously. The study protocol is outlined in Figure 1.

Resting energy expenditure (REE) was measured with the Deltatrac Metabolic Monitor (Datex-Engstrom Division, Helsinki, Finland), calibrated every day. Energy requirement was measured within 24 h before study or control feeding was started. During measurements, nutrition was not interrupted. Body height and weight were (self-)reported at admission. REE was measured for a minimum of one hour. Total energy expenditure (TEE) was calculated by adding 10% (activity factor) above REE [26]. Nutrition was based on TEE and total protein was aimed for at 1.5–1.7 g/kg/day but at least not under 1.2 g/kg/day [27]. To achieve these goals we used the following enteral formulas: Nutrison Protein Plus®, Nutrison Standard® (both from Nutricia, Zoetermeer, Netherlands), and Promote® (Abbott, Columbus, Ohio, US). Data on nutrition and nutritional requirements are listed in Tables 1 and S1 (see Supplementary Material available online at http://dx.doi.org/10.1155/2016/1373060).

Baseline characteristics and routine clinical blood variables were documented. APACHE II (Acute Physiology and Chronic Health Evaluation) scores were calculated as measures of severity of disease in ICU patients. A baseline blood sample was taken for amino acid concentration analysis.

All patients received stable isotopes both enterally and intravenously, on separate days (days 3 and 4, in random order). The enteral tracers were coadministered through a separate port on the tube; the intravenous tracers were administered in the antecubital vein. After 3 days of glutamine enriched or control feeding, if patients were considered stable, at approximately 10.00 am, an arterial baseline sample was collected to measure natural background enrichment followed by a primed continuous intravenous or enteral tracer infusion in random order. Blood samples were collected at 30-minute intervals for 2.5 hours. The same protocol ran the following day with the alternative route of tracer administration. In case stability of the patient was not guaranteed or clinical situation did not allow research, the tracer protocol was postponed by a maximum of one day.

Blood was collected in prechilled heparinized vacuum tubes (BD Vacutainer, Franklin Lakes, NJ) and immediately placed on ice. Blood was centrifuged (10 minutes, 3000 rpm, 4°C) and plasma was extracted and again centrifuged (10 minutes, 3000 rpm, 4°C) after which 500 *μ*L of plasma was added to 20 mg dry sulfosalicylic acid (Across Inc., Geel, Belgium) to precipitate plasma proteins. After vortex mixing, deproteinized plasma samples were snap-frozen in frozen carbon dioxide and stored at −80°C until assayed.

### 2.3. Stable Isotopes

Stable isotope tracers of L-[2-^15^N]-glutamine, L-[5-^13^C-4,4,5,5^2^H_4_]citrulline, and L-[guanidino^15^N_2_]-arginine were used to investigate the effect of the enteral supplementation of glutamine on the metabolism of L-glutamine, L-citrulline, and L-arginine, as well as the conversions of L-glutamine into L-citrulline and L-citrulline into L-arginine. Tracers will be noted as glutamine [M + 1], citrulline [M + 5], and arginine [M + 2], respectively. Tracers were purchased from Cambridge Stable Isotope Laboratory (Woburn, MA, USA). The Department of Clinical Pharmacy at the Erasmus Medical Center in Rotterdam, Netherlands, prepared sterile and pyrogen-free stock solutions of the tracers. The glutamine tracer was prepared 1-2 days before tracer infusion, due to the limited stability of glutamine in solution (72 h). The stock solutions were diluted with physiological saline solution minutes before the start of the tracer administration.

The tracers were administered intravenously and enterally, to study splanchnic extraction of glutamine and to distinguish between the contribution of endogenous and exogenous L-glutamine to the metabolic interrelationship between L-glutamine, L-citrulline, and L-arginine.

Tracers and amounts are listed in Table 2. Tracer dosages were calculated by using previous results from Ligthart-Melis et al. and van de Poll et al. [24, 28]. Because these studies involved surgical patients in the postabsorptive state, a pilot was performed within the first two patients to confirm steady state within our continuously enterally fed critically ill patients [29]. This resulted in a small body weight related adjustment of the priming dose.

### 2.4. Laboratory Analyses

Amino acid concentrations in plasma and infusates were measured using high-performance liquid chromatography, as described elsewhere [30]. Isotopic enrichment was expressed as tracer to tracee (labeled versus unlabeled substrate) ratio (TTR, %), corrected for contribution of lower masses and for background TTR (determined in the baseline sample). Glutamine, citrulline, and arginine TTRs were measured by liquid chromatography-mass spectrometry [30].

### 2.5. Calculations

Isotopic enrichment was adjusted for natural enrichment and for the contribution of overlapping isotopomer distributions of the tracee and tracers with lower masses to the measured TTR as described by Vogt et al. [31]. Metabolic conversions were calculated using established calculations [32]. Since all tracers were administered during enteral nutrition (no matter which route of administration), adjustments were made for tracee infusion, as explained beneath in the calculations and also used by Buijs et al. [33].

For each amino acid studied, arterial enrichment curves were fitted for each patient with the use of PRISM software (version 4.03; GraphPad Software Inc., San Diego, CA). Steady state was calculated by curve fitting plateau calculations. Primarily a first-order straight line was calculated (mean minus baseline). Hereafter an exponential decay function challenged the null hypothesis (first-order straight line), when steady state was in fact more likely to have optimized following a plateau after correction for possible occurring under- or overpriming (this would be a line that decays to a plateau with a constant rate *K*).

The plasma rate of appearance (WBRA: *μ*mol*∗*kg^−1^ *∗*h^−1^) of glutamine, citrulline, and arginine and the known infusion rate of these tracers are based on the following equation:

(1) $$\begin{array}{c} WBRA=\frac{I\left(tracer\right)}{TTR}. \end{array}$$

*I*(tracer) is the known infusion rate of the tracers and TTR is the tracer/tracee ratio. Knowing that enteral feeding and alanyl-glutamine infusion affect the RA, the WBRA calculation includes the exogenous infusion rate of tracee:

(2) $$\begin{array}{c} WBRA=RA\left(\;endogenous\;\right)+I\left(\;tracee\;\right). \end{array}$$

*I*(tracee) reflects the exogenous amino acid (AA) supply (amino acids given by enteral nutrition):

(3) $$\begin{array}{c} I\left(\;tracee\;\right)=I\left(\;AA\;\right)*\left[\;\frac{TTR\left(EN\right)}{TTR\left(IV\right)}\;\right], \end{array}$$

with TTR(EN) being the TTR with enterally administered tracers and TTR(IV) the TTR with intravenously administered tracers, corrected for splanchnic extraction of AA, reflected by splanchnic tracer extraction.

True RA (RA(endogenous)) is therefore calculated as follows [33]:

(4) RAendogenous=ItracerTTRIV−IAA∗TTRENTTRIV.

Calculation of the rate of WB plasma turnover (*Q*: *μ*mol*∗*kg^−1^ *∗*h^−1^) of glutamine into citrulline was performed by using the following equation from Castillo et al. adjusted for endogenous RA [13]:

(5) Q glncit=RAendogenousCIT∗TTR  CIT  M+1TTR  GLN  M+1,

where WBRACIT is the plasma WBRA of citrulline, calculated from the TTR of the infused CIT M + 5 tracer by using (1), and CIT M + 1 is the CIT M + 1 coming from GLN M + 1.

Likewise, calculation of the WB plasma turnover of citrulline into arginine (de novo synthesis) was performed by using the following equation:

(6) Q citarg=RAendogenousARG∗TTR  ARG  M+5TTR  CIT  M+5,

where WBRaARG is the WBRa of arginine, calculated from the TTR of ARG M + 1 by using (1), and ARG M + 5 is the ARG M + 5 coming from CIT M + 5.

Splanchnic extraction (%) of glutamine, citrulline, and arginine was calculated as follows:

(7) $$\begin{array}{c} \left[\;1-\left(\;\frac{TTR\left(EN\right)}{TTR\left(IV\right)}\;\right)\;\right]*100. \end{array}$$

### 2.6. Statistical Analyses

Data are expressed as mean ± standard error (SEM) in case of normally distributed data and as median ± interquartile range (IQR) when data were not normally distributed (tested by Shapiro-Wilk normality test). Independent sample *t*-test or Mann-Whitney test was used to compare control group with alanyl-glutamine group, according to distribution.

One sample *t*-test was used to test whether steady state of metabolic products differed from zero. Plasma values over time were compared using ANOVA and Bonferroni to determine specific time differences.

A *p* value of <0.05 (2-tailed) was considered as statistically significant. Statistical analysis was performed with SPSS 17.0 for Windows® (SPSS Inc., Chicago, IL, USA).

## 3. Results

Twenty patients were successfully included: ten received enteral alanyl-glutamine isocalorically and isonitrogenous enteral nutrition compared to 10 control patients. Results of one patient (CON) were not completely obtained due to detubation and subsequent removal of enteral tube at the last day. One patient got discharged and had his parenteral tracer administration accidently interrupted (ALA-GLN); therefore only enteral results could be obtained. Patient characteristics are summarized in Table 3. Baseline characteristics were not significantly different when comparing the two groups.

### 3.1. Nutrition

Energy expenditure was similar in both groups. All patients received 100% of the caloric target during the tracer infusions. During the whole study period 16 out of 20 received an actual nutrition of >97% of the aimed 5-day nutrition (tube feeding stopped or was lowered during the 5-day course), one patient received 89%, one 80%, one 70%, and one 66%. The last patient was eventually excluded due to unobtained steady state (described below). Mean energy intake during complete study period was 94.8% (SE 2.3) of target nutrition.

Nutrison Standard (lowest protein content) was used more often in patients in the alanyl-glutamine group, because the formula was chosen based on total amount of nitrogen (within the enteral formula) adjusted to the administered amount of alanyl-glutamine.

Although the alanyl-glutamine group received slightly more nitrogen, nitrogen per kg bodyweight was similar. Glutamine administration was as expected significantly higher in the alanyl-glutamine group. Citrulline administration was absent in both groups and total administered arginine was not different in both groups (Table 4).

### 3.2. Glutamine, Citrulline, and Arginine Metabolism

Alanyl-glutamine was not detectable in arterial plasma in both groups. Arterial plasma concentrations of glutamine, citrulline, and arginine were not significantly different at D0 for both groups. Plasma concentrations of glutamine and arginine did not differ during the study period or between groups. Plasma concentrations of citrulline increased significantly from baseline compared to 3-4 days in the control group (D0: 28 ± 4 *μ*mol/mL, D3: 34 ± 3.3 *μ*mol/mL, D4: 39 ± 5.5 *μ*mol/mL, *p* = 0.035), without differences between groups.

In one patient, an isotopic steady state for glutamine, citrulline, and arginine tracers could not be reached with intravenous administration; therefore these results were excluded from analyses. This patient appeared to have higher bilirubin and creatinine levels (although not above exclusion level). Apart from this mentioned patient, steady state for the amino acid tracers could not be calculated for arginine M + 2 (EN: 1 case) and arginine M + 1 (EN: 2 cases). These results were therefore excluded from analyses as well. Steady state curves are presented in Figure S1.

TTR% for almost all infused tracers were higher when intravenously administered compared to enteral infusion, in both groups (Table 5). TTR% of infused tracers was not significantly different between control and alanyl-glutamine group. TTR% of metabolic products differed in case of citrulline M + 1 for both the intravenous and enteral experiments (Table 5).

The TTR% of the metabolic products of [15N]glutamine metabolism—[15N]citrulline and [15N]arginine—were significantly different from zero in both groups with either way of administration. However, TTR% of the metabolic product of L-[5-^13^C-4,4,5,5^2^H_4_]citrulline metabolism, [5-^13^C-4,4,5,5^2^H_4_]arginine, was below detection level in 6 and 7 control patients (iv and enteral tracer administration, resp.) and 4 and 3 patients in the alanyl-glutamine group (iv and enteral tracer administration, resp.).

Endogenous rates of appearance were not significantly different for all administered tracers in the alanyl-glutamine group as compared to the control group (Table 5).

**Figure 2:**
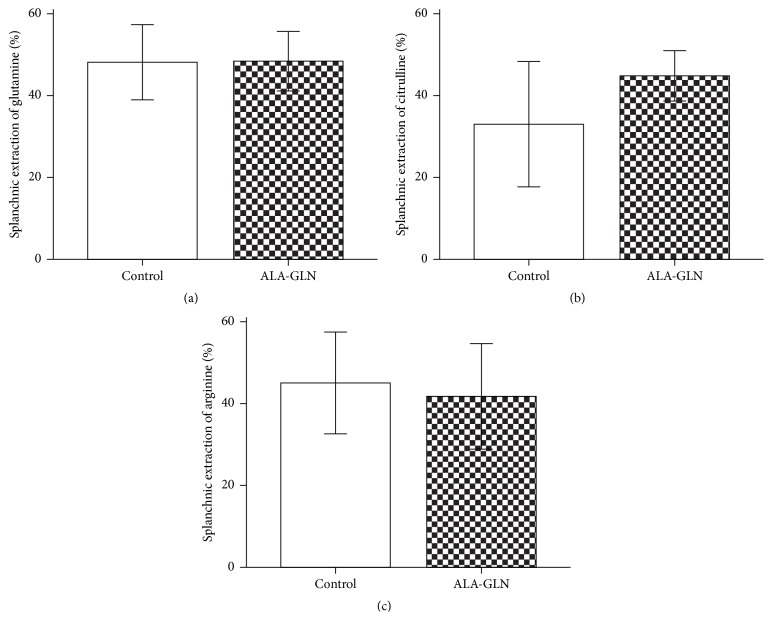
Splanchnic extraction: splanchnic extraction of glutamine (a), citrulline (b), and arginine (c) expressed in mean ± SE.

Splanchnic extraction of glutamine and citrulline was not significantly different for both groups (Figure 2).

**Figure 3:**
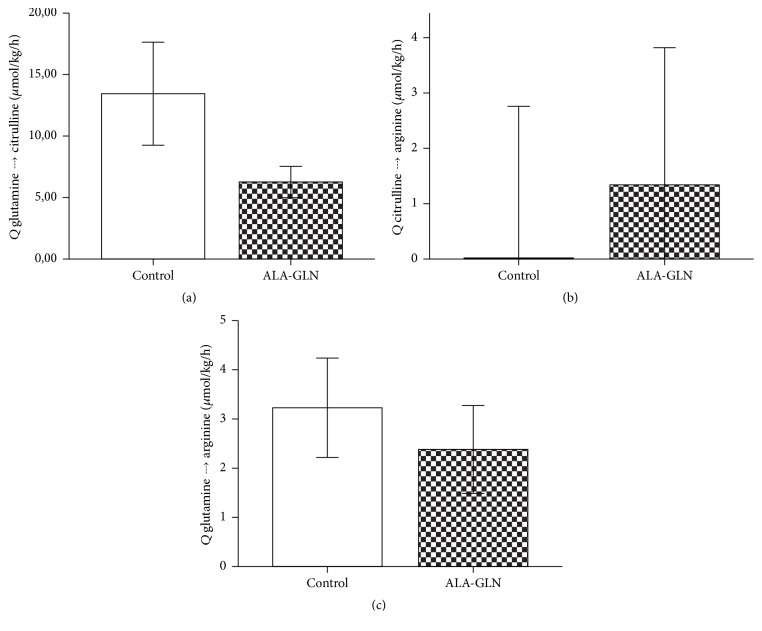
Conversion of glutamine into citrulline and arginine. Conversion rates in *μ*mol/kg/h of glutamine into citrulline (a), citrulline into arginine (b), and glutamine into arginine (c). (a) and (c) are expressed in mean ± SE and (b) is expressed in median ± 95% CI.

Whole body plasma turnover gln → cit (*Q*: *μ*mol*∗*kg^−1^ *∗*h^−1^) was not significantly higher in glutamine-administered patients. In contrast, in control patients, 47.8% (±7.8) of the citrulline was derived from glutamine, versus 24.8% (±4.4; *p* = 0.018) in the alanyl-glutamine group. The percentage of citrulline that served as substrate for arginine was 0% (range 0–10.8) versus 6.5% in the alanyl-glutamine group (range 1.3–12.5 ns). The percentage of glutamine that was converted into arginine was 1.3% (range 1.0–1.4) versus 0.7% (range 0.3–1.3, ns) (Table 5 and Figure 3).

## 4. Discussion

The primary aim of the present study was to quantify the effect of a therapeutically relevant dose of enteral L-glutamine on the synthesis of L-citrulline and subsequent L-arginine in critically ill patients receiving enteral nutrition. In contrast with our working hypothesis, we did not demonstrate a significantly higher turnover of glutamine into the substrates citrulline and arginine in this group.

Glutamine is one of the most abundant amino acids in the human body. In healthy adults, the small intestine is the major organ of glutamine utilization. Enterocytes extract both arterial glutamine and in a greater extent luminal glutamine.

Intestinal glutamine degradation starts with deamination into glutamate and ammonia. Ammonia is released into the portal vein, after which it can be taken up by the liver serving ureagenesis and glutamine synthesis. Glutamate is released into the portal vein, either as glutamate, as alanine, or as *α*-ketoglutarate after transamination with pyruvate, or it is converted to citrulline (approximately 12%, which is 60%–80% of the total citrulline) in which the amino-group and the carbon skeleton of the original glutamine molecule are preserved. The major part of this citrulline is released into the portal vein and subsequently taken up by the proximal tubular cells of the kidney for arginine de novo synthesis [22].

Citrulline itself is scarce in a regular human diet. It was first identified in the 1930s and its name is based on the juice of watermelon (*Citrullus vulgaris*) [34]. In contrast to other amino acids, it is not used in protein synthesis, so it was long time considered to function solely as a metabolic intermediate, specifically in the urea cycle. However, with extensive research performed over the past decades, citrulline appears to play a considerable role in the regulation of nitrogen homeostasis and in the cardiovascular system as regulator of immunity.

After hepatic escape and renal extraction, an ammonia group from aspartate is incorporated to form arginine (catalysed by argininosuccinate synthase [ASS] and argininosuccinate lyase [ASL]) in the proximal convoluted tubules of the kidney. Citrulline is the only precursor for de novo arginine synthesis, of which the majority is executed in the kidneys. This pathway is called the “intestinal-renal axis.” The synthesized arginine from citrulline accounts for 60% of the de novo whole body arginine synthesis; however this only represents 5%–15% of the total circulating arginine [35]. This indicates that most of the plasma arginine is derived from proteolysis and food intake. This arginine pool is sufficient to provide the body's full arginine requirements in physiological conditions.

The relationship between glutamine and arginine has been subject of research in our group since the early nineties [1, 36]. Since then, extensive research has been evolved on using tracer methodology on this topic by us and others. Although the existence of the relationship between glutamine, arginine, and citrulline is clear, we learned that (A) mice metabolism is unequal to human metabolism [35, 37, 38], (B) enteral glutamine administration does not have the same effect as intravenous glutamine supply [23], and (C) critically ill patients behave differently as opposed to healthy volunteers [39–41]. At least three matters have remained unclear: Does postabsorptive glutamine handling differ from the postprandial state? Do critically ill patients metabolize an additional enteral dose of glutamine differently than (so far investigated) trace dosages? And do differences exist between septic and relatively stable ICU patients? We attempted to provide the answers to the first two questions. Kao et al. and Luiking et al. investigated amino acid metabolism in septic ICU patients using stable isotope methodology. Kao et al. show an altered glutamine metabolism in fasted septic patients compared to healthy volunteers [39]. With enteral administration, they show a more pronounced glutamine to citrulline conversion, as was observed earlier in non-critically ill patients [23]. Both investigators observed diminished de novo arginine synthesis. These findings strongly suggest that arginine availability is indeed at risk in septic patients [40, 42].

Since Heylands recent publication on glutamine and antioxidant supplementation in critically ill patients, concerns were raised about glutamine supplementation within their study population [10]. It has now been argued that safety is not guaranteed when high dosages (0.35 g/kg/d parenterally and 30 g/d enterally) of glutamine are administered to patients with multiple organ failure. Given the fact that liver and/or kidney failure impairs protein clearance, glutamine is probably best given to either surgical or medical critically ill patients but should not be given in case of liver or kidney failure [43, 44].

Our results could be explained by a number of considerations as follows. Primarily, the patients were well fed and not glutamine, citrulline, or arginine deficient. Therefore the use of additional glutamine may not have been as effective as within truly depleted patients. Attributing to this, most severely ill (and possibly most depleted) patients could not be included, due to the five-day study period in which dropout must be avoided.

Secondly, due to adapted nutritional formulas, control patients received an average of 10.4 grams of glutamine per day. Given the equal amounts of glutamine splanchnic extraction rates and the equal endogenous rates of appearances, the gut does not seem to metabolize glutamine differently when it comes to different amounts of enteral delivery.

The absence of glutamine promoting arginine synthesis was unexpected. In fact, control patients had a relatively larger glutamine into citrulline conversion rate (13.4 versus 6.3 *μ*mol/kg/h) with significantly higher CIT M + 1 TTR% in the control group. Since glutamine and citrulline compete for the same transporter (neutral amino acid system N transporter: SN1), similar or more citrulline splanchnic extraction can be explained with little glutamine supply [45, 46]. Some studies have previously demonstrated the capability of the liver to take up citrulline [28]. However, this uptake was associated with a release of the liver as well, so unidirectional uptake was never demonstrated.

Unfortunately, due to study design, we were unable to provide any insight neither on hepatic versus intestinal nor on renal citrulline metabolism.

Remarkably, in our experiments, citrulline to arginine turnover and glutamine to arginine turnover were lower compared to Ligthart-Melis, with a conversion rate of 0–6.5% and 0.7–1.3%, respectively, differing with a factor of 5–10% compared to earlier experiments. Again, splanchnic extraction and enteral administration partly account for this.

Remarkably, in the control group, the median conversion of citrulline to arginine was calculated zero while having higher glutamine to arginine conversion rates. This is because the TTR% of the metabolic product of L-[5-^13^C-4,4,5,5^2^H_4_]citrulline metabolism, [5-^13^C-4,4,5,5^2^H_4_]arginine, was below detection level in most of the control patients. The glutamine to arginine production probably finds its origin in the gut by the enzymes argininosuccinate synthase and argininosuccinate lysase. Circumstantial induction seems evident since this has been subject of discussion earlier [24, 47].

Furthermore, as we know renal citrulline metabolism is autoregulated [21]: in the presence of adequate arginine concentrations, arginine de novo synthesis is diminished, whereas at low concentrations renal arginine de novo synthesis is promoted. The faith of the “unused” citrulline is unclear, but it can be used for many systems.

Most importantly, our experiments prove that excessive arginine production after glutamine supplementation does not occur; hence the safety of 0.5 g/kg/day enteral alanyl-glutamine administration is proven in relatively stable critically ill patients without sepsis, kidney, or liver failure.

### 4.1. Methodological Perspective

Glutamine stable isotope studies have generated intense debate recently, since Marini et al. and Tomlinson et al. published multiple tracer results in mice and fed volunteers, respectively [38, 48]. Marini et al. found discrepancies between nitrogen and carbon labeled glutamine in mice implying that glutamine provides nonspecific carbon for the citrulline ureido group. Tomlinson et al. found overestimation of the glutamine contribution to arginine synthesis with the labeled nitrogen and found a contribution of 56% when using labeled carbon (compared to the 64% found earlier in fasted surgical patients). In the light of the first study and earlier published results it can be concluded that there are interspecies differences. With Tomlinson et al.'s study, the overestimation of recycling tracers due to splanchnic extraction remains quantitatively unclear, because no correction was made with whole body rates of appearances calculated solely with intravenous tracers. Our results show 48% glutamine splanchnic extraction, with lower enterally administered glutamine M + 1 TTR%, resembling results of Bourreille et al. [49] and lower glutamine to citrulline conversion rates (24.8–47.8%). Importantly the glutamine systemic delivery (endogenous infusion rate) after splanchnic extraction and corrected for steady state nutrient (tracee) delivery was not different in both groups. In contrast RA (not corrected) with enteral administration almost doubled intravenous administration (Table 5). Therefore, overestimation due to splanchnic extraction is proven by our experiments and future tracer studies should not use the dilution equations on solely enteral tracer experiments, as also addressed by Ligthart-Melis and Deutz [50]. This also implies that when correctly using the dilution equations it is still not definite which glutamine tracer should best be used for future studies. We suggest additional research on this topic using transition LC-MS/MS enabling differentiation between different fragments of the labeled amino acids; however this method includes similar quantitative pitfalls. A multistep approach with multilabeled amino acids could probably be the golden standard, but then inevitable setting associated bias (as discussed below) also disqualifies this approach.

### 4.2. Strength and Limitations of the Study

The cross-over study design enabling correcting for splanchnic extraction and enteral feeding is a strength although it can also be seen as a weakness. Although patients were considered stable, within the ICU stability and clinical condition of patients can vary every minute. Therefore an approach with two separate study days does not cover small metabolic changes that may have occurred in the meantime. Additionally, different metabolic phases with different energy needs are observed within this patient population [27]. The initial metabolic phase after administration was covered by the three-day administration of TEE-based nutrition with or without glutamine. Furthermore, by randomizing the administration order we attempted to outbalance potential metabolic differences. An alternative approach in which simultaneous administration through both routes is studied has the disadvantage of different tracer usage (often giving rise to different metabolic outcome) or (when given sequentially on the same day) different timing within circadian rhythm.

The study design disqualified the use of a control group, due to a five-day continuous enteral feeding regime while being immobilized to mimic minimal basal energy expenditure.

The heterogeneity of the studied population means that interpretation should be with caution. It also means that this is a reflection of the exact population that is able to receive full enteral nutrition: no instability, no bowel surgery, no sepsis, and no expected quick discharge. Therefore these results are useful as a pilot for larger investigation on enterally enriched nutrition.

Unfortunately two patients could not fulfil their second tracer study day. This is a risk that goes hand in hand with the clinical setting. Stable isotope studies are usually performed with 5-6 patients (per group), due to complexity and expenses of the method.

In conclusion, these results prove that, in critically ill nonseptic patients receiving optimal enteral nutrition including a clinically relevant dose of glutamine, the relationship between glutamine, citrulline, and arginine is still present. However in the glutamine receiving group there was no extra citrulline or arginine synthesis and splanchnic glutamine extraction was not increased. Arginine synthesis was not promoted by glutamine administration indicating that in this population glutamine supplementation is safe. This also suggests that for arginine synthesis enhancement there is no need for an additional dose of glutamine when these patients are adequately fed. Furthermore, we proved that overestimation of calculated metabolic products can be reduced by correcting for splanchnic extraction and enteral nutrition.

## Supplementary Material

Table S1 displays complete amino acid content and composition of the different enteral formulas used, expressed in g/L.
Figures S1A, S1B, and S1C show steady states achieved for GLN M+1, CIT M+5 and ARG M+2. Figures show TTR% in CON and ALA-GLN (DIPEP); when enterally and intravenously administered.

## Figures and Tables

**Table 1:** Enteral nutrition.

Enteral nutrition	Energy (kcal/L)	Protein (g/L)	Glutamine (g/L)	Citrulline (g/L)	Arginine (g/L)
Nutrison Standard	1000	40 g/L	4.6	0	1.6
Nutrison Protein Plus	1250	63 g/L	7.19	0	2.5
Promote	1000	63 g/L	2.3	0	2.3

**Table 2:** Tracer dosages.

Tracer	Prime (*µ*mol/kg)		Infusate (*µ*mol/kg/h)	
	CON mean (SEM)	ALA-GLN mean (SEM)	CON mean (SEM)	ALA-GLN mean (SEM)
GLN M + 1	232.0 (13.8)	236.7 (8.0)	18.4 (1.6)	16.0 (1.5)
CIT M + 5	627.2 (37.3)	639.8 (21.8)	1.2 (0.3)	1.0 (0.3)
ARG M + 2	141.1 (8.4)	144.0 (4.9)	1.3 (0.1)	1.2 (0.1)

**Table 3:** Baseline characteristics.

Baseline parameter	CON	ALA-GLN
	*N* (%)/mean (SEM)/median (IQR)	*N* (%)/mean (SEM)/median (IQR)
Demographics		
Sex: male/female (%)	6/4 (60/40)	6/4 (60/40)
Age (y)	65 (6.4)	57 (5.4)
Length (cm)	172.6 (3.7)	176.8 (3.0)
Weight (kg)	73.2 (5.8)	77.7 (4.1)
BMI (kg/m^2^)	24.2 (1.0)	24.5 (3.3)
*Clinical assessment*		
Type of ICU admission		
Respiratory insufficiency	6 (60)	3 (30)
Cardiogenic shock	1 (10)	4 (40)
Neurotrauma	1 (10)	0
Multitrauma	1 (10)	2 (20)
Other	1 (10)	1 (10)
APACHE II score	27.0 (2.2)	25.3 (3.0)
Laboratory measurements at inclusion		
pH	7.42 (0.048)	7.44 (0.027)
pCO_2_	41.4 (10.3)	43.9 (9.7)
Bicarbonate (mmol/L)	*32.9* (*22.0–33.0*)	30.5 (5.3)
Glucose (mmol/L)	6.6 (0.83)	7.5 (1.4)
Leukocytes (10^9^ *µ*mol/L)	11.0 (3.1)	11.8 (4.4)
Bilirubin (*µ*mol/L)	*8.5* (*5.0–16.8*)	9 (4.0–11.3)
Creatinine (*µ*mol/L)	78 (9.7)	81.8 (13.7)
Urine production (mL/24 h)	2301 (361)	2333 (295)

**Table 4:** Nutrition characteristics.

Nutrition parameter	CON mean (SEM)	ALA-GLN mean (SEM)	Sig. between groups (*p*)
Calorimetry			
REE (kcal/24 h)	1847 (165)	1998 (85)	0.429
TEE (kcal/24 h)	1998 (165)	2176 (80)	0.350
VCO_2_ (mL/min)	223 (17.0)	234 (10.7)	0.587
VO_2_ (mL/min)	269 (24.8)	292 (12.4)	0.577
RQ	0.84 (0.03)	0.81 (0.016)	0.237
Nutrition			
Nutrison Protein Plus/Nutrison Standard/Promote	3/5/2 (30/50/20)	0/1/9 (0/10/90)	
Received % of nutritional target (study period)			
Median (IQR)	98.5 (84.3; 100)	100 (98.8–100)	0.136
Energy (kcal/d)	1999 (165)	2190 (88)	0.325
Received energy (kcal/d)	1844 (183)	2129 (67)	0.170
Nitrogen			
(g/d)	101.5 (7.2)	122.7 (5.8)	0.034
(g/kg/d)	1.41 (0.08)	1.59 (0.11)	0.066
Received nitrogen			
(g/d)	93.1 (7.9)	119.4 (5.1)	0.014
(g/kg/d)	1.30 (0.11)	1.54 (0.02)	0.055
Received glutamine			
(g/d)	10.2 (1.0)	35.2 (0.17)	(*<0.000*)
(mmol/kg/d)	0.98 (0.09)	3.11 (0.03)	(*<0.000*)
Citrulline (g/d)	—	—	—
Received arginine			
(g/d)	3.6 (0.32)	3.2 (0.13)	0.278
(mmol/kg/d)	0.29 (0.08)	0.24 (0.01)	0.066
Baseline plasma glutamine (*µ*mol/mL)	521 (66)	497 (37)	0.743
D3	539 (21)	518 (34)	0.592
D4	525 (32)	510 (38)	0.755
Baseline plasma citrulline (*µ*mol/mL)	28 (4)	32 (3)	0.365
D3	33 (3)	37(2)	0.349
D4	39 (5)	38 (3)	0.865
Baseline plasma arginine (*µ*mol/mL)	53 (6)	72 (8)	0.076
D3	64 (6)	62 (6)	0.833
D4	68 (7)	64 (5)	0.645

**Table 5:** Tracer dynamics.

Tracer parameters and calculations	CON mean (SEM)/median (IQR)	ALA-GLN mean (SEM)/median (IQR)	Difference CON versus ALA-GLN (*p*)
*Glutamine*			
RA GLN M + 1			
IV	364.8 (57.5)	390.4 (40.0)	0.720
EN	787.5 (91.6)	606.3 (88.3)	0.173
Endogenous RA GLN	335.3 (62.3)	322.04 (39.4)	0.856
TTR% GLN M + 1			
IV	4.88 (0.38)	4.88 (0.33)	0.997
EN	2.54 (0.35)	2.44 (0.25)	0.820
Splanchnic extraction GLN	48.2 (4.6)	48.4 (3.6)	0.965
*Citrulline*			
RA CIT M + 5			
IV	26.5 (4.7)	28.5 (5.5)	0.780
EN	51.7 (23.7; 69.2)	29.5 (20.8; 64.3)	0.462
Endogenous RA CIT	26.5 (4.7)	28.5 (5.5)	0.780
TTR% CIT M + 1			
IV	1.99 (0.33)	1.13 (0.16)	***0.031***
EN	4.44 (0.47)	2.64 (0.46)	***0.015***
TTR% CIT M + 2			
IV	0.29 (0.14)	0.21 (0.08)	0.622
EN	1.08 (0.21)	0.50 (0.19)	0.057
TTR% CIT M + 5			
IV	4.31 (0.85)	4.06 (0.34)	0.791
EN	2.49 (2.25; 2.94)	1.98 (1.82; 2.29)	0.207
Splanchnic extraction CIT	33.0 (7.7)	44.8 (3.1)	0.185
*Arginine*			
RA ARG M + 2			
IV	76.6 (12.2)	75.0 (6.0)	0.907
EN	135.4 (106.8; 240.3)	121.3 (17.0)	0.374
Endogenous RA ARG	26.9 (25.0; 58.0)	31.8 (6.1)	0.336
TTR% ARG M + 1			
IV	0.44 (0.13)	0.35 (0.09)	0.583
EN	0.97 (0.25)	0.83 (0.15)	0.624
TTR% ARG M + 2			
IV	6.33 (0.76)	6.00 (0.50)	0.717
EN	3.73 (0.78)	3.26 (0.46)	0.593
TTR% ARG M + 5			
IV	0.33 (0.06)	0.35 (0.07)	0.898
EN	0.25 (0.05)	0.30 (0.05)	0.500
Splanchnic extraction ARG	45.0 (6.2)	41.8 (6.4)	0.725
*Conversion rates*			
*Q* Gln → Cit	13.4 (4.2)	6.3 (1.3)	0.121
*Q* Cit → Arg	0 (0–2.2)	1.3 (0.5–3.1)	0.135
*Q* Gln → Arg	3.2 (1.0)	2.4 (0.9)	0.539
*Q* Gln → Cit% of Gln	4.45 (0.91)	2.06 (0.42)	0.026
*Q* Gln → Cit% of Cit	47.8 (7.8)	24.8 (4.4)	0.018
*Q* Cit → Arg% of Cit	0 (0–10.8)	6.5 (1.3–12.5)	0.370
*Q* Cit → Arg% of Arg	0 (0–7.4)	5.3 (2.2–10.4)	0.131
*Q* Gln → Arg% of Gln	1.3 (1.0–1.4)	0.7 (0.3–1.3)	0.370

RA in *µ*mol/kg/h, endogenous RA in *µ*mol/kg/h, splanchnic extraction in %, TTR in %, and *Q* in *µ*mol/kg/h.

## Abbreviations

NO: Nitric oxide
REE: Resting energy expenditure
TEE: Total energy expenditure
APACHE II: Acute Physiology and Chronic Health Evaluation II
TTR: Tracer to tracee ratio
(WB)RA: (Whole body) rate of appearance
*I*: Infusion rate
AA: Amino acid
*Q*: Plasma turnover
CON: Control group
ALA-GLN: Group receiving alanyl-glutamine.
