# Supplementary material for: Enteral Glutamine Administration in Critically Ill Nonseptic Patients Does Not Trigger Arginine Synthesis
Source: J Nutr Metab. 2016 Apr 20;2016:1373060. doi: 10.1155/2016/1373060 (PMC4855021; doi:10.1155/2016/1373060)
Supplement: Supplementary file 1 — Table S1 displays complete amino acid content and composition of the different enteral formulas used, expressed in g/L. Figures S1A, S1B, and S1C show steady states achieved for GLN M+1, CIT M+5 and ARG M+2. Figures show TTR% in CON and ALA-GLN (DIPEP); when enterally and intravenously administered. [file 1373060.f1.pdf]

Supplemental table S1.

Amino Acid Concentration (g/l)	Nutrison Standard®	Nutrison Protein Plus®	Promote®
Glutamine	4.6	7.2	2.3
Citrulline	0	0	0
Arginine	1.6	2.5	2.3
Alanine	1,4	2,1	1,8
Asparaginezuur	3,1	4,9	2,7
Cystine	0,1	0,2	0,3
Glycine	0,8	1,3	1,2
Histidine	1,3	2,0	1,6
Isoleucine	2,3	3,6	2,9
Leucine	4,2	6,6	5,5
Lysine	4,1	6,4	4,6
Methionine	1,3	2,1	1,6
Phenylalanine	2,2	3,5	3,0
Proline	4,0	6,3	6,3
Serine	2,6	4,1	3,4
Threonine	2,0	3,1	2,6
Tryptofaan	0,6	0,9	0,7
Tyrosine	2,4	3,8	3,2
Valine	2,9	4,5	3,7

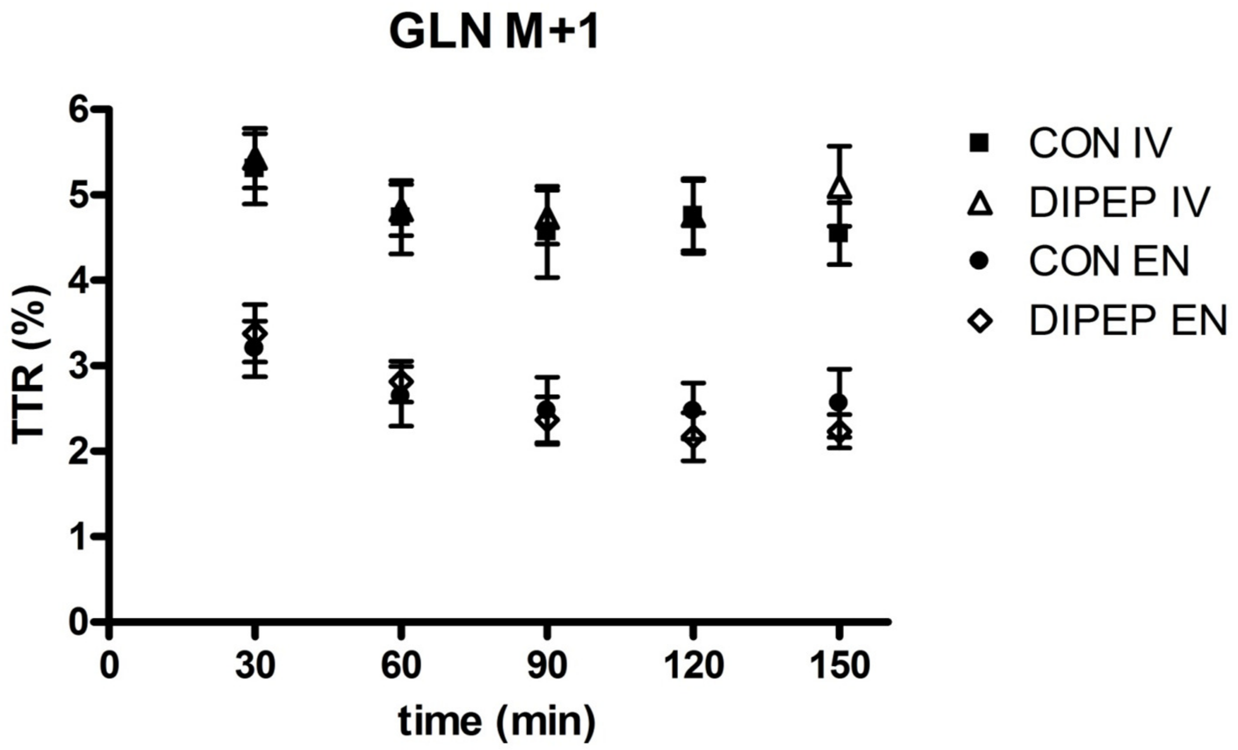

GLN M+1
TTR (%)
time (min)
0
1
2
3
4
5
6
0
30
60
90
120
150
CON IV
DIPEP IV
CON EN
DIPEP EN

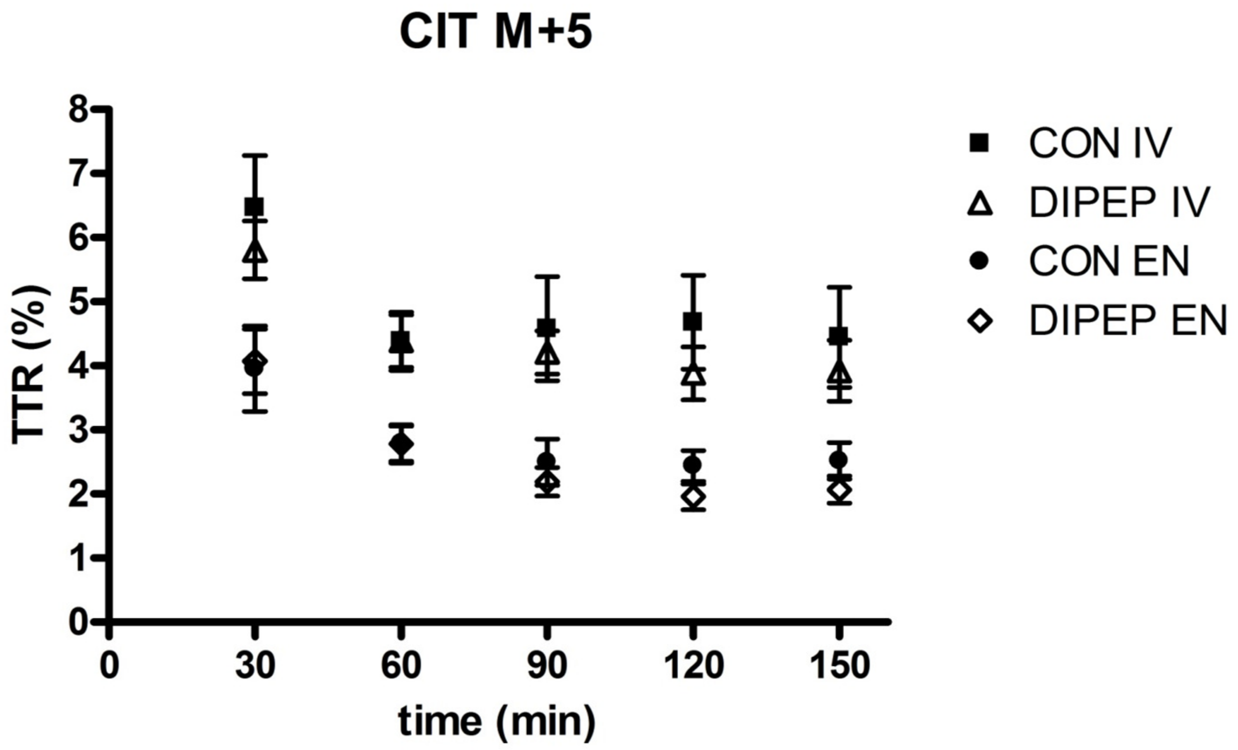
CIT M+5
TTR (%)
8
7
6
5
4
3
2
1
0
0
30
60
90
120
150
time (min)
CON IV
DIPEP IV
CON EN
DIPEP EN

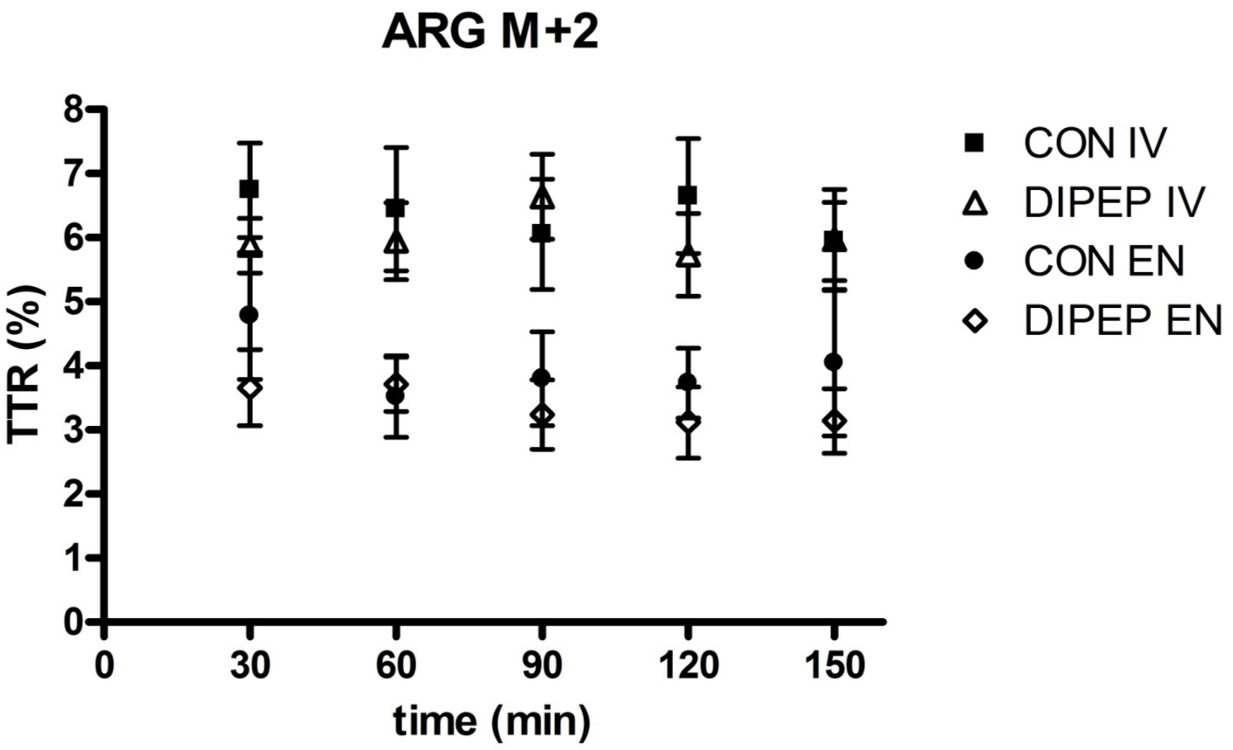
ARG M+2
TTR (%)
0
1
2
3
4
5
6
7
8
0
30
60
90
120
150
time (min)
CON IV
DIPEP IV
CON EN
DIPEP EN
